# 供者T细胞Stat3基因敲除对小鼠急性肠道移植物抗宿主病的影响

**DOI:** 10.3760/cma.j.cn121090-20250107-00011

**Published:** 2025-04

**Authors:** 玉溪 许, 筱淇 王, 世杰 杨, 清晓 宋, 锦 魏, 曦 张

**Affiliations:** 1 川北医学院附属医院血液内科，南充 637002 Department of Hematology, Affiliated Hospital of North Sichuan Medical College, Nanchong 637002, China; 2 陆军军医大学第二附属医院血液病医学中心，血液生态与智慧细胞科学创新中心，全军临床重点专科，创伤与化学中毒国家重点实验室，血液病与微环境重庆市重点实验室，重庆 400037 Medical Center of Hematology, Xinqiao Hospital of Army Medical University, Blood Ecology and Smart Cell Science Innovation Center, State Key Laboratory of Trauma and Chemical Poisoning, Chongqing Key Laboratory of Hematology and Microenvironment, Chongqing 400037, China; 3 金凤实验室，重庆 401329 Jinfeng Laboratory, Chongqing 401329, China

**Keywords:** 急性移植物抗宿主病, 肠道损伤, Stat3, T细胞, 肠道干细胞, 肠道类器官, Acute graft-versus-host disease, Gastrointestinal injury, Stat3, T cells, Intestinal stem cells, Intestinal organoids

## Abstract

**目的:**

探讨供者T细胞Stat3基因敲除对急性肠道移植物抗宿主病（GI-aGVHD）的影响及其机制。

**方法:**

对BALB/c小鼠进行清髓剂量辐照，经尾静脉输注BALB/c小鼠（同基因对照组）、C57BL/6小鼠（野生型T细胞组，WT组）及C57BL/6J-Stat3^em1cyagen^小鼠（Stat3基因敲除T细胞组，Stat3-KO组）的骨髓和脾脏细胞构建aGVHD模型。监测小鼠生存率、体重变化及临床评分，流式微球阵列术检测血清细胞因子浓度，分离组织中的淋巴细胞进行流式细胞术分析，HE染色后观察肠道病理学变化，FITC-葡聚糖检测肠道通透性，免疫组化评估Ki67和Muc2的表达，实时荧光定量逆转录PCR（qRT-PCR）分析小肠Olfm4、Lysozyme和Muc2的基因表达水平，代谢组学检测血清和肠道代谢物。同时，构建小肠类器官与T细胞的共培养体系，体外模拟GI-aGVHD模型，观察类器官数量与面积变化。此外，通过接种荧光素酶转染的急性淋巴细胞白血病（ALL/Luc）细胞和生物发光成像来评估移植物抗白血病（GVL）效应。

**结果:**

与WT组相比，Stat3-KO组小鼠临床症状（体重下降、弓背、腹泻）较轻，生存率较高（*P*<0.05），IL-2、IL-6、IFN-γ、TNF-α、IL-17A以及IL-10血清浓度较低（均*P*<0.05），肠道炎症细胞浸润及肠黏膜通透性减弱（均*P*<0.05）。此外，Stat3-KO组小肠Muc2和Ki67表达显著上调（均*P*<0.05），Olfm4、Lysozyme和Muc2基因的表达水平亦明显上调（均*P*<0.05）。体外实验显示，Stat3-KO组的类器官发育优于WT组。代谢组学分析提示，敲除供者T细胞Stat3基因减轻GI-aGVHD可能与调节胆汁酸及不饱和脂肪酸代谢相关。在GVL小鼠模型中，回输去除T细胞的骨髓细胞（TCD-BM组）的ALL/Luc细胞迅速生长，而Stat3-KO组未观察到肿瘤生长，80％小鼠的无肿瘤存活期超过100 d（*P*<0.05）。

**结论:**

敲除供者T细胞Stat3基因可减轻T细胞对肠道干细胞的损伤，从而缓解GI-aGVHD的肠道损伤，同时保留稳定的GVL效应。

## Abstract (en)

**Objective:**

To investigate the effects and underlying mechanisms of Stat3 knockout in donor T cells on acute gastrointestinal graft-versus-host disease（GI-aGVHD）.

**Methods:**

BALB/c mice were exposed to lethal irradiation and transplanted with bone marrow and spleen cells from BALB/c mice (syngeneic control group), C57BL/6 mice (wild-type T cell group, WT group), or C57BL/6J-Stat3^em1cyagen^ mice (Stat3 gene knockout T cell group, Stat3-KO group) via tail vein injection to establish the aGVHD model. The survival rate, body weight changes, and clinical scores of mice were monitored. Cytometric bead array (CBA) was used to detect the concentrations of serum cytokines. Lymphocytes were isolated from tissues for flow cytometric analysis. H&E staining was performed to observe intestinal pathological changes. FITC-dextran assay was conducted to assess intestinal permeability. Immunohistochemistry was used to evaluate the expression of Ki67 and Muc2. Real-Time Quantitative Reverse Transcription PCR (qRT-PCR) was employed to analyze the gene expression levels of Olfm4, Lysozyme, and Muc2 in the small intestine. Metabolomics was conducted to detect metabolites in serum and intestinal tissues. An in vitro GI-aGVHD organoid model was established by coculturing intestinal organoids with allogeneic T cells, where the number and area of small intestinal organoids were recorded. The GVL effect was assessed using luciferase-transfected ALL cells（ALL/Luc）and bioluminescent imaging.

**Results:**

Compared with the WT group, Stat3 knockout T cells alleviated body weight loss, reduced symptoms—such as hunchback and diarrhea—in mice, improved survival rate（*P*<0.05）, and reduced serum interleukin（IL）-2, IL-6, interferon-γ, tumor necrosis factor-α, IL-17A, and IL-10 levels（all *P*<0.05）, intestinal inflammatory cell infiltration（*P*<0.05）, and intestinal mucosal permeability. Further, Muc2 and Ki67 expression levels in the small intestine of the Stat3 knockout group were markedly increased, and Olfm4, Lysozyme, and Muc2 gene expression levels were significantly increased（all *P*<0.05）. In vitro, the Stat3 knockout group demonstrated better organoid development than the WT group. Metabolomic analyses indicated that Stat3 knockout in T cells may affect the pathways associated with bile acid secretion and unsaturated fatty acids. ALL/Luc cells in the GVL mouse model proliferated rapidly in the TCD-BM group; however, 80% of the mice in the Stat3-KO group survived tumor-free for >100 days（*P*<0.05）.

**Conclusion:**

Knocking out Stat3 in graft T cells reduces T cell damage to intestinal stem cells, thereby ultimately alleviating GI-aGVHD while maintaining a stable GVL effect.

异基因造血干细胞移植（allo-HSCT）是治疗血液恶性肿瘤的有效手段[1]。急性移植物抗宿主病（aGVHD）是allo-HSCT后最常见且致死率较高的并发症[2]–[3]，其中肠道aGVHD（GI-aGVHD）是导致移植后早期死亡的主要原因[4]–[5]。研究表明，GI-aGVHD的发生与异基因反应性T细胞对肠道干细胞（ISC）的攻击密切相关[6]–[7]，可导致肠道上皮损伤修复能力显著下降[8]。因此，保护ISC是治疗GI-aGVHD的核心策略之一。

aGVHD发病机制涉及多方面，主要包括预处理引发组织损伤，释放炎性细胞因子、活化受者抗原提呈细胞，促使供者T细胞活化扩增，同时募集中性粒细胞，最终导致皮肤、肝脏和肠道的急性损伤。目前，大多数研究认为aGVHD主要是由异基因反应性T细胞介导[9]。其中，异基因T细胞的内在信号活动起关键作用。转录因子Stat3在免疫调控和炎症反应中扮演重要角色，其异常活化不仅与多种恶性肿瘤相关[10]–[12]，还与T细胞谱系分化及功能密切相关[13]。以往研究显示，Stat3基因通过抑制初始T细胞活化和调节炎症因子分泌，能够显著减轻GVHD的严重程度[14]–[17]。此外，Stat3基因对ISC的生长和存活至关重要[18]，敲除Stat3基因的肠上皮中分离出的隐窝无法体外培养成肠道类器官[19]。已知供者T细胞介导的ISC损伤是GI-aGVHD的主要发病机制[20]，但是T细胞介导的ISC损伤是否与Stat3信号通路相关尚不明确，靶向供者T细胞的Stat3基因是否能减轻其对ISC的损伤从而减轻GI-aGVHD，目前缺乏直接证据。

近年来，类器官技术的快速发展，为肠道疾病机制研究提供了新平台。肠道类器官源于ISC的体外培养[21]–[22]，具备与体内高度相似的微绒毛和肠隐窝结构，为研究ISC在GI-aGVHD中的作用提供了重要工具。此外，代谢组学分析作为揭示疾病代谢通路变化的有力手段，已显示出其在GVHD研究中的应用潜力。已有研究提示，胆汁酸及不饱和脂肪酸代谢紊乱与GI-aGVHD的发生密切相关[23]–[26]，探索供者T细胞Stat3基因敲除对胆汁酸及不饱和脂肪酸代谢的调控作用，可为研究GI-aGVHD的发生机制提供新的视角。

基于此，本研究通过构建aGVHD动物模型和体外肠道类器官模型并结合代谢组学分析，系统探讨敲除供者T细胞Stat3基因对GI-aGVHD的保护作用及其潜在机制。

## 材料与方法

1. 实验动物：8～12周SPF级雄性C57BL/6小鼠和BALB/c小鼠购自北京维通利华实验动物技术有限公司，C57BL/6J-Stat3^em1cyagen^小鼠购自赛业（苏州）生物科技有限公司。所有小鼠均饲养于陆军军医大学实验动物中心SPF级动物培养室，且所有实验方案均经陆军军医大学新桥医院机构动物保护与使用委员会批准。

2. 试剂与仪器：肠道类器官完全培养基和温和细胞解离剂（加拿大StemCell公司产品）；基质胶（美国Corning公司产品）；DPBS（美国Hyclone公司产品）；无菌1×PBS（Solarbio公司产品）；RPMI 1640培养基和DMEM/F-12培养基（VivaCell公司产品）；Calcein/PI细胞活性与细胞毒性检测试剂盒（碧云天生物技术有限公司产品）；细胞活性检测试剂盒（伯桢生物科技有限公司产品）；Olfm4、Lysozyme及辣根过氧化物酶山羊抗兔二抗（美国CST公司产品）；CD90.2 MicroBeads和MACS细胞分选仪（德国Miltenyi Biotec公司产品）；FITC-葡聚糖（美国Sigma公司产品）；RNAfast200 total RNA快速提取试剂盒（飞捷生物技术公司产品）；PrimeScript™ FAST RT试剂盒（日本Takara公司产品）；Cytometric Bead Array（CBA）Mouse Th1/Th2/Th17 CBA试剂盒、CD16/32、CD4、H-2k^b^、IFN-γ和Foxp3购自美国BD公司；CD8、IL-17、IL-4、IL-5、IL-13、TNF-α、qua viability dye、Foxp3/转录因子流式固定破膜缓冲液和500×细胞刺激剂购自美国Thermo Fisher Bioscience公司；TCR-β、IFN-γ、H-2k^d^、GM-CSF购自美国Biolegend公司。

3. aGVHD模型的建立：BALB/c受体小鼠分两次接受共750 cGy的全身照射。将供体小鼠5×10^6^个骨髓细胞和2.5×10^6^个脾细胞经尾静脉注入受鼠体内。每周观察2～3次，并采用先前描述的评分系统[27]进行aGVHD临床评分。

4. 移植物抗白血病（GVL）效应模型的建立：在aGVHD模型的基础上，尾静脉注射1×10^4^个荧光素酶转染的急性淋巴细胞白血病（ALL/Luc）细胞，并间隔5 d进行生物发光成像。

5. 流式微球阵列术（CBA）：眼眶取血后，室温静置1 h，12 000×*g*离心5 min，取血清，存放于−80 °C。后续检测按照CBA试剂盒说明书进行。

6. 病理评分：组织标本经甲醛固定、石蜡包埋、切片及HE染色后，用Olympus光学显微镜拍摄图像。根据前述的评分系统[28]评估病理严重程度。

7. 免疫组化：切片在染色前烘片30 min、脱蜡、柠檬酸缓冲液修复抗原，3％过氧化氢水溶液阻断。Muc2（1∶2000）和Ki67（1∶400）一抗4 °C孵育过夜，洗涤后加入二抗，孵育1 h后进行DAB染色、脱水封片、成像及统计分析。

8. FITC-葡聚糖检测肠道通透性：FITC-葡聚糖用PBS配成40 mg/ml工作浓度。小鼠断食4 h后进行灌胃，每只400 µl。4 h后采集外周血，常温静置1 h，3000×*g*离心10 min，取上清，12 000×*g*、4 °C离心10 min。取100 µl上清与等量PBS加入96孔板，检测自发荧光值（*A*_485/535_）。

9. 细胞因子流式细胞术分析：提取各靶器官淋巴细胞，用含10％ FBS的RPMI 1640培养基重悬并加入1×细胞刺激剂，于孵箱中培养4 h。然后按照Foxp3/转录因子流式固定破膜缓冲液说明书进行细胞因子染色。

10. T细胞的分选：取小鼠脾脏，用70 µm细胞过滤器研磨。用CD 90.2 MicroBeads对T细胞进行阳选，再通过MACS细胞分选仪获得T细胞，将其悬浮于含10％FBS的RPMI 1640培养基中。

11. 小肠类器官的培养及传代：小肠隐窝的提取、培养及传代参照Stemcell肠道类器官培养说明书进行。

12. 体外肠道GVHD类器官模型的构建：将分选后的T细胞加入1×细胞刺激剂，置于37 °C孵箱刺激2 h。刺激结束后，4 °C、500×*g*离心5 min。去除上清液，用肠道类器官完全培养基重悬沉淀，沿侧壁加入孔板。类器官种板数量：96孔板50个，48孔板200个，24孔板400个，类器官与T细胞的比例为1∶2000。

13. 小肠类器官活性鉴定：小肠类器官培养7 d后转至离心管，常温静置5 min后移除上清。加入1 ml预冷的PBS重悬，200×*g*、4 °C离心5 min。根据说明书，加入适量的Calcein AM/PI检测工作液，再种回24孔板中，37 ºC避光孵育30 min。孵育完成后，在荧光显微镜下观察染色效果（Ex/Em＝535/617 nm）。

14. 小肠类器官HE染色及免疫荧光染色：小肠类器官培养7 d后转至离心管，常温静置5 min后移除上清。加入1 ml预冷的PBS重悬，再次静置5 min。尽量去除上清，保留沉淀。加入4％多聚甲醛于4 °C固定样品2 h。弃上清，留沉淀进行脱水、石蜡包埋、3 µm连续切片，进行HE染色和免疫荧光染色。

15. 实时荧光定量逆转录PCR（qRT-PCR）检测Olfm4、Lysozyme和Muc2基因mRNA表达：RNA提取、逆转录、实时荧光定量PCR操作参考试剂盒说明。引物序列均由北京擎科生物科技股份有限公司合成与纯化（表1）。

**表1:** 实时荧光定量逆转录PCR检测Olfm4、Lysozyme和Muc2基因的引物序列

基因	引物序列（5′→3′）	
	正向	反向
Muc2	GTGAAGACCGAGATTGTG	TGGCACTTGTTGGAATAC
Olfm4	AGTACACAGCTCACATCCTTTCT	TCTCCTTCACTTCCAGCTTGATC
Lysozyme	AAGGAATGGAATGGATGGCTACC	TTGATCCCACAGGCATTCTTAGA
GAPDH	CATCACTGCCACCCAGAAGACTG	ATGCCAGTGAGCTTCCCGTTCAG

16. 统计学处理：采用GraphPad Prism 9.5软件进行统计分析与作图。计量资料以“均值±标准误”表示。死亡率比较采用Log-rank检验。两组间比较采用非配对*t*检验，三组及以上比较采用one-way ANOVA分析，实验至少重复3次。*P*<0.05为差异具有统计学意义。

## 结果

一、敲除供者T细胞Stat3基因对小鼠aGVHD的影响

构建aGVHD模型，设立同基因对照组、回输野生型T细胞组（WT组）和Stat3敲除T细胞组（Stat3-KO组）。移植后30 d每2～3 d观察、记录1次小鼠体重及临床症状评分。在体重变化方面，移植早期三组体重均下降，同基因对照组随后回升，WT组持续下降，而Stat3-KO组在第7天起体重开始回升。临床症状方面，WT组在移植后第3天起逐渐出现弓背、耸毛、活动度降低及持续性腹泻等aGVHD症状且逐渐加重；Stat3-KO组在移植早期也出现上述表现，但症状较轻，偶尔腹泻但持续时间不超过3 d，随后症状消失。因此其临床评分显著降低，小鼠腹泻发生率较低（*P*<0.05，图1A、1B）。生存率方面，WT组小鼠在10 d内全部死亡，而同基因对照组和Stat3-KO组均存活至移植后30 d，总体生存率明显高于WT组（均*P*<0.05，图1C）。

**图1:**
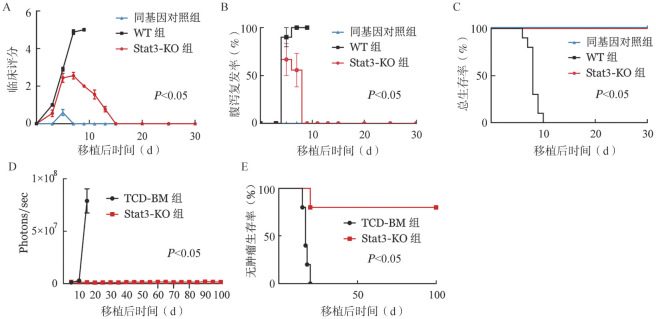
敲除供者T细胞Stat3基因对小鼠急性移植物抗宿主病（aGVHD）和移植物抗白血病（GVL）效应的影响（A～C，*n*＝10；D、E，*n*＝5） **A** aGVHD临床评分；**B** 腹泻发生率；**C** 总生存曲线；**D** photons/second曲线；**E** 无肿瘤生存曲线 **注** WT组：回输野生型T细胞；Stat3-KO组：回输Stat3基因敲除T细胞；TCD-BM组：回输去除T细胞的骨髓细胞

为了评估敲除供者T细胞Stat3对GVL效应的影响，设立两组分别回输去除T细胞的骨髓细胞（TCD-BM组）和Stat3敲除T细胞（Stat3-KO组），移植时通过尾静脉注射接种1×10^4^个ALL/Luc细胞，构建GVL小鼠模型。TCD-BM组的ALL/Luc细胞迅速生长，20 d内均死亡。而Stat3-KO组有80％小鼠无肿瘤存活超过100 d（*P*<0.05，图1D、E）。

二、敲除供者T细胞Stat3基因对小鼠血清炎性细胞因子浓度和T细胞亚群的影响

以往研究显示T细胞亚群分化与aGVHD密切相关[29]，而Stat3基因是调控T细胞亚群分化的重要因素[30]。为了探究敲除供者T细胞的Stat3基因对T细胞亚群的影响，收集移植后第7天小鼠的血清样本，进行流式细胞术检测（图2A）。与同基因对照组相比，WT组小鼠IL-2、IL-6、IFN-γ、TNF-α、IL-17A以及IL-10血清浓度均显著升高（均*P*<0.05，图2B～H）。与WT组相比，Stat3-KO组小鼠IL-2、IL-6、IFN-γ、TNF-α、IL-17A以及IL-10血清浓度显著降低，三组间IL-4血清浓度差异无统计学意义（图2B～H）。于移植后第7天，分离小鼠脾脏、肝脏、肺和小肠的淋巴细胞并进行流式细胞术分析。与WT组相比，Stat3-KO组小鼠的肝脏和肺中IL-17^+^、GM-CSF^+^的CD4^+^和CD8^+^ T细胞比例降低，小肠中IFN-γ^+^、IL-17^+^和TNF-α^+^ CD4^+^ T细胞比例降低（图3A～L）；在脾脏中，IFN-γ^+^和TNF-α^+^ CD4^+^ T细胞比例显著降低（图3A、G），IL-17^+^的CD4^+^和CD8^+^ T细胞比例降低（图3E、F）。在脾脏、肝脏、肺和小肠中，Foxp3^+^的CD4^+^及CD8^+^的调节性T细胞（Treg）比例均有显著升高（图3K、L）。与IFN-γ^+^ CD4^+^ T细胞（Th1）不同，IFN-γ^+^CD8^+^ T细胞（Tc1）在脾脏、肝脏和肠道中没有明显差异，Stat3-KO组在肺中轻度增高（图3A、B）。IL-4/5/13^+^CD4^+^T细胞（Th2）细胞在脾脏中明显减少，肺中明显增高，肝脏和肠道没有显著性差异（图3C），IL-4/5/13^+^CD8^+^T细胞（Tc2）在各个组织器官均无显著性差异（图3D）。

**图2:**
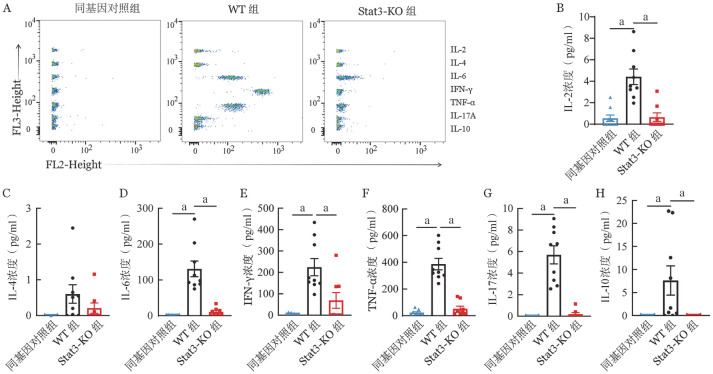
敲除供者T细胞Stat3基因对移植后小鼠血清炎性细胞因子浓度和T细胞亚群的影响（*n*＝8～10，a：*P*<0.05） **A** 流式微球阵列术（CBA）检测血清IL-2、IL-4、IL-6、IFN-γ、TNF-α、IL-17A以及IL-10浓度的流式示意图；**B～H**分别为各组IL-2、IL-4、IL-6、IFN-γ、TNF-α、IL-17A以及IL-10血清浓度比较 **注** WT组：回输野生型T细胞；Stat3-KO组：回输Stat3基因敲除T细胞

**图3:**
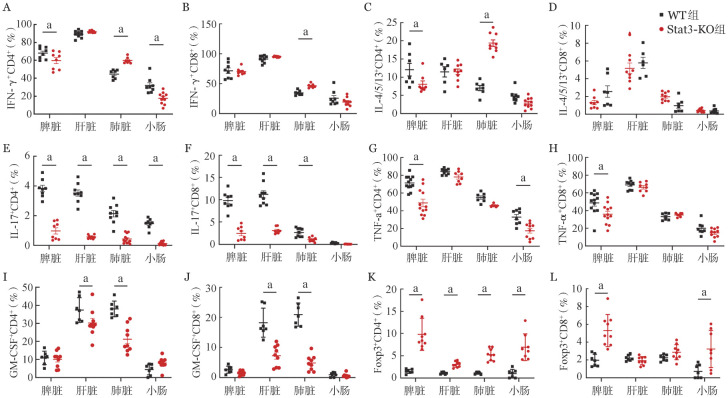
敲除供者T细胞Stat3基因对移植后小鼠血清炎性细胞因子浓度和T细胞亚群的影响（*n*＝8～10，a：*P*<0.05） **A** 各器官IFN-γ^+^ CD4^+^细胞百分比；**B** 各器官IFN-γ^+^ CD8^+^细胞百分比；**C** 各器官中IL-4/5/13^+^ CD4^+^细胞百分比；**D** 各器官IL-4/5/13^+^CD8^+^细胞百分比及急性GVHD临床评分；**E** 各器官IL-17^+^ CD4^+^细胞百分比；**F** 各器官IL-17^+^CD8^+^细胞百分比；**G** 各器官TNF-α^+^ CD4^+^细胞百分比；**H** 各器官TNF-α^+^ CD8^+^细胞百分比；**I** 各器官GM-CSF^+^ CD4^+^细胞百分比；**J** 各器官GM-CSF^+^ CD8^+^细胞百分比；**K** 各器官Foxp3^+^ CD4^+^细胞百分比；**L** 各器官Foxp3^+^ CD8^+^细胞百分比 **注** WT组：回输野生型T细胞；Stat3-KO组：回输Stat3基因敲除T细胞

上述结果提示，敲除供者T细胞的Stat3基因可抑制Th1和Th/Tc17、扩增Treg，显著减轻aGVHD，同时保留GVL效应。

三、敲除供者T细胞Stat3基因对小鼠GI-aGVHD的影响

为进一步评估肠道组织损伤程度，于移植后第7天采集小肠和结肠组织样本进行HE染色（图4A）。与同基因对照组相比，WT组小鼠的小肠和结肠可见炎症细胞浸润、黏膜下层间隙增大、隐窝坏死和绒毛结构破坏现象，组织病理评分显著升高。而Stat3-KO组与WT组相比，其小肠和结肠炎症细胞浸润较轻，绒毛和隐窝结构完整，组织病理评分显著降低（*P*<0.05，图4A、B）。同时，在移植后第7天，WT组和Stat3-KO组小鼠口服FITC标记的葡聚糖，4 h后Stat3-KO组血清中FITC-葡聚糖含量显著低于WT组（*P*<0.05，图4C）。以上结果表明，敲除供者T细胞Stat3基因可减少炎症细胞的组织浸润及其对肠上皮细胞的损伤、降低肠黏膜通透性。

**图4:**
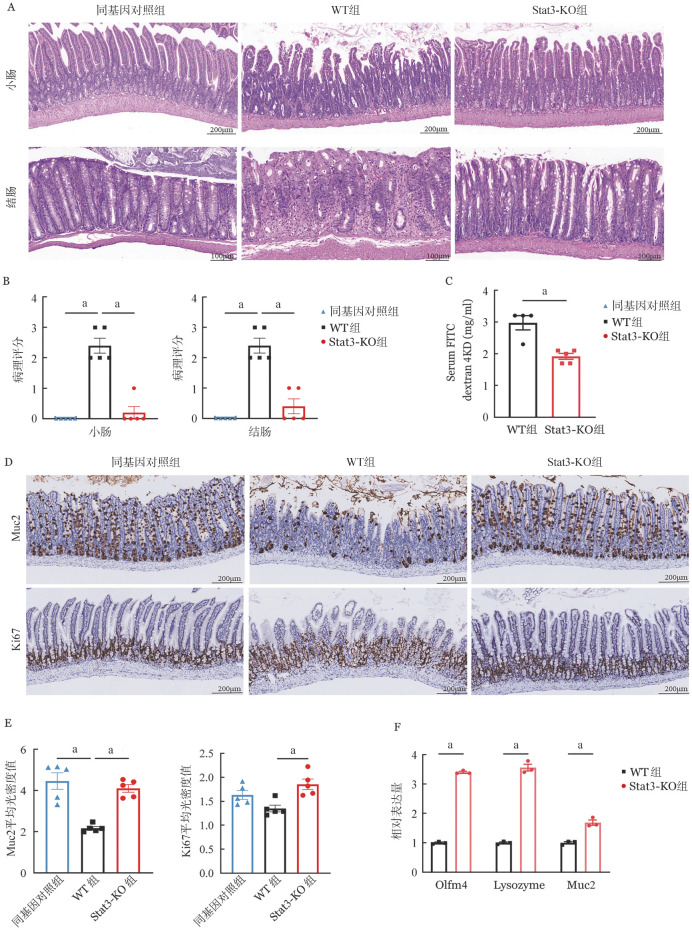
敲除供者T细胞Stat3基因对小鼠急性肠道移植物抗宿主病的影响（a：*P*<0.05） **A** 小肠和结肠的病理变化（HE染色）；**B** 病理评分结果；**C** 血清FITC-葡聚糖水平（*n*＝4～5）；**D** Muc2和Ki67的小肠免疫组化染色；**E** 免疫组化评分结果（*n*＝3～5）；**F** 小肠组织qRT-PCR结果（*n*＝3～5） **注** WT组：回输野生型T细胞；Stat3-KO组：回输Stat3基因敲除T细胞

移植后第7天收集小鼠小肠组织，进行Muc2和Ki67免疫组化染色。与同基因对照组相比，WT组小鼠Muc2表达量显著降低，提示GI-aGVHD小鼠伴有Muc2^+^杯状细胞减少（图4D）；与WT组相比，Stat3-KO组小鼠Muc2表达量明显增高且与同基因对照组相近（图4D）。这些结果表明移植Stat3-KO T细胞可以维持肠道黏液屏障功能（*P*<0.05，图4E）。其次，Stat3-KO组Ki67表达升高且与WT组差异有统计学意义（*P*<0.05，图4E），提示Stat3-KO T细胞可能对肠上皮细胞增殖有促进作用。此外，与WT相比，Stat3-KO组的Olfm4（ISC标记物）、Lysozyme（潘氏细胞标记物）和Muc2（杯状细胞标记物）基因表达均显著上调（均*P*<0.05，图4F）。

四、BALB/c小鼠肠道类器官的培养与鉴定

肠道类器官是体外评估T细胞对ISC损伤的最有效方法之一[6]，为进一步评估敲除T细胞的Stat3对ISC的影响，我们首先建立肠道类器官模型。图5A显示了单个隐窝细胞发育为小肠类器官的生长进程，7 d左右成熟且为传代的最佳时机。取第7天成熟的肠道类器官进行鉴定。可见肠道类器官活性良好（图5B）；HE染色可见肠道类器官结构完整，形态正常（图5C）；免疫荧光可见Olfm4（ISC标记物）和Lysozyme（潘氏细胞标记物）表达（图5D）。以上结果表明BALB/c小鼠肠道类器官构建成功。

**图5:**
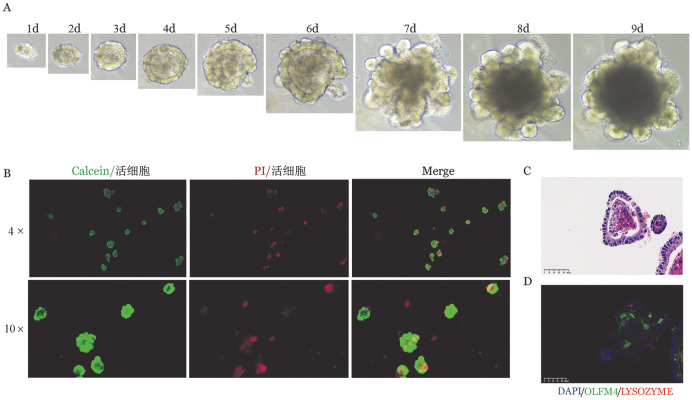
BALB/c小鼠肠道类器官的培养与鉴定 **A** BALB/c小鼠肠道类器官生长明场图；**B** BALB/c小鼠肠道类器官第7天Calcein/PI细胞活性检测（4×的标尺为500 µm，10×的标尺为200 µm）；**C** HE染色；**D** 免疫荧光

五、通过体外肠道GVHD类器官模型验证供者T细胞Stat3基因敲除对ISC损伤的影响

小肠类器官稳定传三代后用于模型构建。提取C57BL/6小鼠（WT组）或C57BL/6J-Stat3^em-1cyagen^小鼠（Stat3-KO组）脾脏中的T细胞，加入1×细胞刺激剂于37 °C孵箱刺激2 h。刺激完成后，按T细胞∶小肠类器官2000∶1的比例加入48孔板。在光学显微镜下观察各组小肠类器官生长情况及形态，间隔24 h观察1次。共培养24 h后，WT组已经有小肠类器官开始死亡，Stat3-KO组的类器官形态和数量正常且其面积相较于空白对照组稍大（图6A、B）。48～72 h可见WT组小肠类器官大量死亡，Stat3-KO组的小肠类器官开始出现少量死亡但存活的小肠类器官面积较大、腔体细胞碎片增多（图6C、D）。

**图6:**
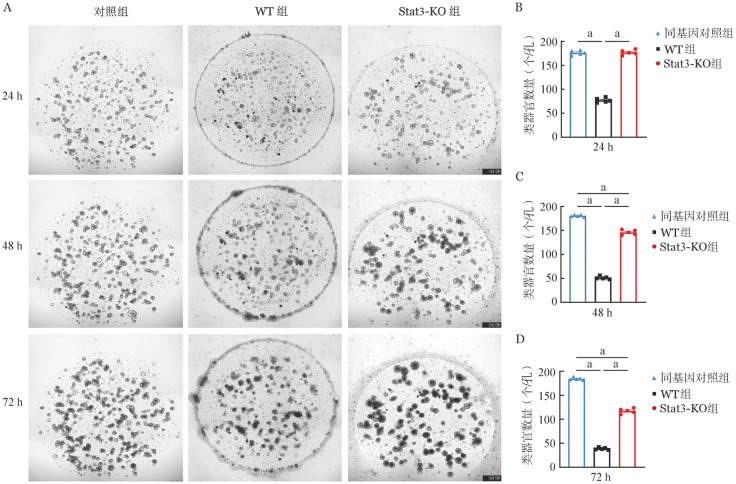
通过体外肠道GVHD模型验证敲除供者T细胞Stat3基因对肠道干细胞损伤的影响 **A** T细胞与BALB/c小鼠肠道类器官共培养24～72 h明场图；**B～D** T细胞与BALB/c小鼠类器官共培养24、48、72 h后各组类器官数量比较（*n*＝5） **注** WT组：回输野生型T细胞；Stat3-KO组：回输Stat3基因敲除T细胞

六、低浓度Stat3基因敲除供者T细胞对ISC生长的影响

上述结果证实敲除Stat3基因可明显减轻T细胞对ISC的损伤，为进一步探索这一效果是否与T细胞数量相关，我们在共培养体系中设置了不同梯度的T细胞数量进行比较，并于48 h记录观察结果。设低（0.1×10^5^个T细胞）、中（0.25×10^5^个T细胞）、高（0.75×10^5^个T细胞）三个浓度梯度组。低浓度下，与空白对照组相比，加入WT-T细胞组的类器官面积及数量明显减少（图7A、B），表明低浓度T细胞即可成功构建体外肠道GVHD模型；而意外的是，Stat3-KO组的肠道类器官面积增大，数量与空白对照组相比无变化，提示低浓度Stat3-KO的T细胞具有促进ISC生长的能力（图7A、B）。中、高浓度时，与空白对照组相比，随着T细胞浓度的升高，WT组的类器官面积及数量进一步降低（图7C、D），中浓度Stat3-KO组的类器官面积及数量无明显变化（图7C）。高浓度时，Stat3-KO组的类器官面积无明显变化，但数量开始减少（图7D），表明敲除Stat3的T细胞减轻T细胞对ISC损伤的效应与T细胞浓度呈负相关。

**图7:**
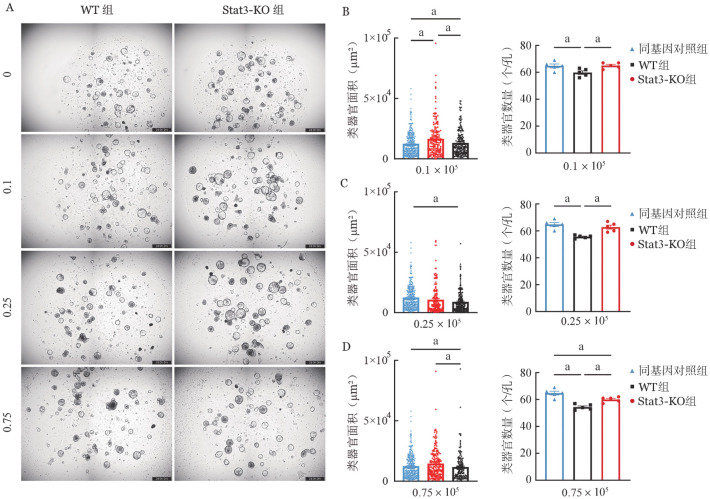
Stat3基因敲除的低浓度供者T细胞对肠道干细胞生长的影响（*n*＝5） **A** 不同数量T细胞（×10^5^）与BALB/c小肠类器官共培养48 h明场图；**B** 低浓度组单个类器官面积变化及数量；**C** 中浓度组单个类器官面积变化及数量；**D** 高浓度组单个类器官面积变化及数量 **注** WT组：回输野生型T细胞；Stat3-KO组：回输Stat3基因敲除T细胞

七、差异代谢物富集分析

构建aGVHD模型，移植后第7天采集小鼠血清及肠道内容物，对差异代谢物进行KEGG富集分析。结果表明敲除供者T细胞的Stat3可能影响移植后胆汁酸分泌、ABC转运、氨基酸代谢和不饱和脂肪酸合成等代谢通路（图8A、B）。这一系列代谢通路的改变可能与Stat3-KO的T细胞减轻肠道损伤的作用密切相关。

**图8:**
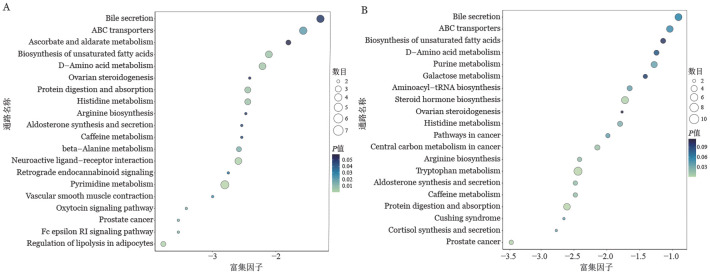
血清及肠内容物的非靶代谢组学的差异代谢物富集分析结果 **A** 血清差异代谢物显著性前20的KEGG富集结果；**B** 肠内容物差异代谢物显著性前20的KEGG富集结果

## 讨论

aGVHD是allo-HSCT后的常见并发症[2]–[3]，重度GI-aGVHD治疗难度大且致死率高[4]–[5]。本研究利用动物模型和肠道类器官模型结合代谢组学分析，系统探讨了敲除供者T细胞Stat3基因对GI-aGVHD的保护作用及其潜在机制。首先，本研究表明敲除供者T细胞的Stat3能够有效减轻GI-aGVHD并保持稳定的GVL效应。接受Stat3基因敲除T细胞移植的小鼠表现为移植后体重下降及弓背、腹泻等症状明显减轻，生存率显著增高。肠道病理显示炎症细胞浸润减少，肠黏膜通透性减低。通过ALL/Luc的荷瘤实验，证实接受Stat3基因敲除T细胞移植的小鼠能够有效清除体内的肿瘤细胞并且在100 d之内未出现肿瘤复发。

我们对敲除供者T细胞Stat3基因减轻GI-aGVHD并保留GVL效应进行了多角度的机制解析。鉴于Stat3对T细胞谱系分化的影响[13],[29]，首先，我们检测了血清中T细胞谱系相关的细胞因子，Stat3敲除显著降低IL-2、IL-6、IFN-γ、TNF-α、IL-17A以及IL-10等细胞因子浓度。进一步通过流式细胞术检测淋巴器官和GVHD靶器官中T细胞亚群的比例，结果显示Stat3敲除显著降低靶器官中IFN-γ^+^、IL-17^+^和TNF-α^+^ CD4^+^ T细胞比例，增加Foxp3^+^ T细胞比例，提示敲除T细胞Stat3基因可抑制Th1及Th17分化并扩增Treg，我们认为这是Stat3基因敲除减轻GI-aGVHD的重要机制之一，也支持了既往研究结果[31]–[32]。其次，我们发现Stat3基因敲除后IFN-γ^+^ CD8^+^ T细胞及IL-4/5/13^+^ T在大多数的器官中比例无明显变化，提示敲除T细胞Stat3基因不影响Tc1及Th/Tc2的分化。以往研究显示Tc1及Tc2均介导GVL效应，而Tc1介导的GVL效应更强[33]。此外，最新的研究表明2型免疫相关的细胞因子（如IL-4）能有效增强CAR-T细胞的抗肿瘤效应[34]–[35]。基于此，我们认为敲除T细胞的Stat3基因保留Tc1及Th/Tc2的数量是其维持稳定而持久的GVL效应的重要机制。

近期也有研究表明敲除Stat3基因减轻aGVHD不仅依赖调节T细胞亚群的分化[36]，因为通过anti-CD25抗体去除Treg并不影响Stat3基因敲除T细胞减轻aGVHD效应[16]，提示敲除T细胞的Stat3基因减轻aGVHD还存在其他重要机制。

类器官技术作为近年来快速发展的体外研究平台，在模拟复杂疾病微环境方面独具优势。肠道类器官能够高度模拟体内肠道组织的结构和功能，包括细胞组成、空间分布以及一些关键的生理功能，这是细胞系模型所无法比拟的[37]。同时，与动物模型相比，肠道类器官模型具有更好的可控性、更高的通量以及更低的成本，能够更精准地研究特定因素在GVHD发病机制中的作用，避免动物个体差异和复杂体内环境的干扰[38]–[39]。有研究通过提取aGVHD模型小鼠血清，并将其加入肠道类器官培养基的方式构建模拟aGVHD肠道损伤的体外模型[40]。本研究通过异基因反应性T细胞与肠道类器官共培养的方式构建了体外GI-aGVHD模型，更真实地模拟体内情况，能动态评估T细胞对ISC的损伤，准确体现ISC的活力及生长情况，为后续深入探究机制提供了关键工具。通过类器官模型，我们发现敲除Stat3减轻了T细胞对ISC的杀伤效应，同时促进了ISC的生长和相关功能细胞（如杯状细胞和潘氏细胞）的恢复。我们认为，这是敲除T细胞Stat3基因减轻GI-aGVHD的另一个重要机制。我们推测敲除Stat3基因的T细胞促进ISC生长的机制可能有以下两点：①Stat1信号可以促进ISC的增殖[41]，敲除Stat3有可能导致Stat1信号上调[42]；②2型免疫也可以促进组织修复[43]，本研究结果显示敲除Stat3促进Th/Tc2细胞分化。

近年来，很多研究已经证实代谢对免疫具有重要影响[44]，胆汁酸在调节肠道微环境、维持黏膜屏障功能方面发挥重要作用[23],[26]。不饱和脂肪酸及胆汁酸代谢被证实与GVHD密切相关[24]–[25]，且研究发现aGVHD患者中存在胆汁酸池耗竭[45]。本研究发现敲除供者T细胞Stat3基因可上调胆汁酸代谢通路及不饱和脂肪酸合成通路，提示敲除T细胞的Stat3减轻GI-aGVHD可能与调节胆汁酸与不饱和脂肪酸代谢有关。以往研究显示胆汁酸可降低FXR转录活性，而细胞内FXR信号影响同种异基因反应性T细胞功能，FXR缺失的供体T细胞可减轻GVHD、减少T细胞浸润和IFN-γ产生[46]，不饱和脂肪酸通过扩增Treg来减轻GVHD[47]–[48]。因此，我们推测敲除T细胞的Stat3通过增高体内胆汁酸水平抑制异基因反应性T细胞功能，并且促进不饱和脂肪酸的合成来扩增Treg，从而减轻GI-aGVHD。这一发现不仅为通过代谢干预策略防治GVHD提供了新的思路，也为胆汁酸及不饱和脂肪酸代谢调控在肠道疾病中的应用奠定了基础。

本研究存在以下局限：①动物模型虽能在一定程度上对GVHD的病理机制进行解析，但其与患者GVHD的复杂程度相比仍存在差异，未来需结合患者样本进一步验证；②敲除T细胞Stat3基因促进ISC生长的具体机制有待阐明；③胆汁酸及不饱和脂肪酸代谢的详细调控机制仍需通过更深入的代谢组学和分子生物学研究加以阐明。

综上所述，本研究系统探讨了供者T细胞Stat3敲除对GI-aGVHD的保护作用及其潜在机制，探究了供者T细胞的Stat3基因对ISC稳态的关键调控作用。本研究结果可为GI-aGVHD的精准治疗和代谢靶向干预提供理论依据。
